# Effects of Three Herbs on Methane Emissions from Beef Cattle

**DOI:** 10.3390/ani10091671

**Published:** 2020-09-16

**Authors:** María Fernanda Vázquez-Carrillo, Hugo Daniel Montelongo-Pérez, Manuel González-Ronquillo, Epigmenio Castillo-Gallegos, Octavio Alonso Castelán-Ortega

**Affiliations:** 1 Faculty of Veterinary Medicine and Animal Science, Universidad Nacional Autónoma de México, Mexico City CP 04510, Mexico; mafervc@comunidad.unam.mx (M.F.V.-C.); epigmeniocastillo@comunidad.unam.mx (E.C.-G.); 2 Faculty of Veterinary Medicine and Animal Science, Instituto Literario 100, Universidad Autónoma del Estado de México, Colonia Centro, Toluca CP 50000, Mexico; hugo_as159@hotmail.com (H.D.M.-P.); mrg@uaemex.mx (M.G.-R.)

**Keywords:** methane, mitigation, beef cattle, *Cosmos bipinnatus*, *Cymbopogon citratus*, *Matricaria chamomilla*

## Abstract

**Simple Summary:**

Cattle represent a significant source of greenhouse gases (GHGs). In 2010, cattle emitted 5.0 gigatons of CO_2_ equivalents globally, which represents about 62% of the livestock sector emissions. Therefore, mitigating GHGs such as methane (CH_4_) originating from the cattle industry, offers an opportunity to reduce GHG emissions and climate change over the short term. Ruminant nutritionists have developed different strategies, which include the use of antibiotics, herbs and chemical compounds, such as nitrate, to manipulate rumen fermentation and reduce CH_4_ emissions. So, the objectives of the present work were to evaluate the in vivo antimethanogenic effects of three herbs: *Cymbopogon citratus* (CC), *Matricaria chamomilla* (MC) and *Cosmos bipinnatus* (CB) on beef cattle fed a high in concentrate diet and the effects of increasing levels of CC on enteric CH_4_ emissions by beef cattle fed a ration low in concentrate. We concluded that CC significantly reduced methane yield (g of CH_4_/kg of DMI) by 33%, CB reduced methane yield by 28%, and MC had no significant effect. In Experiment 2, CC supplemented with 2% of the daily DMI significantly reduced the total daily CH_4_ emissions by 26% without affecting the supply of nutrients to the animal.

**Abstract:**

The objectives of the present work were to evaluate the in vivo antimethanogenic effects of *Cymbopogon citratus* (CC), *Matricaria chamomilla* (MC) and *Cosmos bipinnatus* (CB) on beef cattle fed a high in concentrate diet (forage-to-concentrate ratio [F:C] of 19.4:80.6), and the effects of increasing levels of CC (0%, 2%, 3%, and 4% of the daily DM intake (DMI)) on enteric CH_4_ emissions by beef cattle fed a ration low in concentrate (F:C ratio of 49.3:50.7). Two experiments were conducted to address the objectives. For the first experiment, eight Charolais × Brown Swiss steers distributed in a replicated 4 × 4 Latin square experimental design were used. Four treatments were evaluated: (1) control diet (CO), (2) CO + 365 g dry matter (DM)/d CB, (3) CO + 365 g DM/d MC, (4) CO + 100 g DM/d CC. For Experiment 2, four Charolais x Brown Swiss steers distributed in a single 4 × 4 Latin square design were used. It was concluded that 100 g DM per day CC and 365 g DM per day CB (Experiment 1) reduced CH_4_ yield of beef cattle. In Experiment 2, CC supplementation levels exceeding 2% of DMI reduced daily CH_4_ emissions but at the expense of decreasing digestibility of DM.

## 1. Introduction

There is growing concern worldwide regarding the role that domestic ruminants play in global warming and climate change. Domestic ruminants produce large amounts of greenhouse gases (GHGs), such as methane (CH_4_), which originates from enteric fermentation and the degradation of feces [1,2]. According to the Environmental Protection Agency of the United States of America [3], in 2018, global CH_4_ production from enteric fermentation represented 28% of the methane emitted globally by the agricultural, forestry and other land-use (AFOLU) sector. The AFOLU sector represents 24% of the total GHGs emitted globally.

The United States is the largest producer of beef worldwide and, as a result, beef and dairy cattle contribute to approximately 48% of the US agricultural GHG emissions reported in 2015 [4,5]. Methane is produced in large volumes by cattle, e.g., up to 716 g/d for a dairy cow [6] and up to 372 g/d for beef cow [7]. This gas is 28 times more powerful than CO_2_ in terms of its capacity to cause the greenhouse effect; however, its lifespan in the atmosphere ranges from only 9 to 15 years [2]. The short lifespan of CH_4_ means that it may be possible to mitigate climate change more rapidly by reducing enteric CH_4_ emissions than by reducing CO_2_ emissions because the CO_2_ can remain in the atmosphere for up to 200 years. Therefore, mitigating the CH_4_ from cattle offers an opportunity to reduce GHG emissions and climate change.

Ruminant nutritionists have developed different strategies, which include the use of antibiotics and plant secondary metabolites and other chemical compounds, such as nitrate, to manipulate rumen fermentation, and reduce CH_4_ and nitrogen (N) emissions [8,9]. It is well known that the use of synthetic antibiotics, e.g., monensin, as feed additives is a useful way to reduce energy losses in the form of CH_4_ in ruminants [10]. For example, according to Appuhamy et al. [11], monensin supplementation reduces CH_4_ emissions by 15% in beef cattle. However, such use of antibiotics has caused public concern because of the presence of residues in milk and beef and the increasing resistance of microbes to treatment with antibiotics. Thus, the use of antibiotics for this purpose is banned in some countries. For the last two decades, scientists have been evaluating the potential of natural feed additives such as herbs and plant extracts, which have also been used for centuries for various purposes in human diets. Herbs such as garlic [12] and oregano [13], have been shown to modulate rumen fermentation, improve nutrient utilization, and reduce CH_4_ production in ruminants. Similarly, lemongrass (*Cymbopogon citratus*) and peppermint (Mentha × piperita), when used as feed additives alone or in combination, have been reported to improve production performance and rumen fermentation efficiency in terms of microbial cell synthesis and VFA production, and reduce CH_4_ production [14].

Furthermore, Wanapat et al. [15] demonstrated that 100 g/d *Cymbopogon citratus* (CC) powder enhanced the digestibility of nutrients, the rumen microbial community (by increasing cellulolytic and amylolytic bacteria), and microbial protein synthesis efficiency, thus improving rumen ecology in beef cattle. However, the antimethanogenic effect of CC was not measured in respiration chambers by Wanapat et al. [14], it was estimated on the basis of the VFAs concentration in rumen liquor. Thus, it is necessary to evaluate the antimethanogenic properties of CC and its potential as an antimethanogenic herb in vivo. This herb has been used for many years in folk medicine because of its antiseptic, antifever, antidyspeptic, antioxidant, antinociceptive, carminative and anti-inflammatory effects [16]. It also has febrifuge, analgesic, spasmolytic, antipyretic, diuretic, tranquilizer and stomachic properties [16], but its antimethanogenic properties have not been evaluated in vivo.

Similarly, herbs such as *Cosmos bipinnatus* (CB), a Mexican Asteraceae, have shown antimethanogenic properties in vitro [17] and in vivo in dairy cattle [18] but have never been tested in beef cattle, particularly those fed diets with high concentrate levels. Additionally, plants such as *Matricaria chamomilla* [MC] have never been evaluated in vivo, despite their high contents of flavonoids and other phenolic compounds, which have been identified in various parts of the MC flower [19]. Flavonoids such as quercetin have been shown to reduce the total population of protozoa and methanogens. In the case of methanogenic bacteria, flavonoids inhibit the synthesis of the bacterial cell wall, the cytoplasmic membrane, and nucleic acid synthesis, which is reflected in a decrease in CH_4_ production [20]. Using real-time PCR amplification, Oskoueian et al. [20] demonstrated that naringin and quercetin significantly reduced the total population of ruminal protozoa by reducing the efficiency of protozoa protein synthesis. Additionally, flavonoids improve fermentation efficiency by increasing propionate production to the detriment of acetate production, thus decreasing the population of methanogenic archaea. Using the in vitro gas production technique, Petrič et al. [19] investigated the effects of four individual medicinal plants, namely, wormwood, chamomile, fumitory, and mallow, and their mixture used as dietary substrates on ruminal and intestinal fermentation parameters, the total ciliate protozoan population, and total antioxidant capacity of rumen fluid. The authors concluded that the mixture of wormwood, chamomile, fumitory and mallow possessed a strong ruminal antioxidant capacity and showed the potential to reduce ruminal and intestinal CH_4_ emissions and ammonia concentrations.

Therefore, the objectives of the present work were to evaluate in vivo the antimethanogenic effects of *C. citratus*, *M. chamomilla* and *C. bipinnatus* in beef cattle fed a finishing diet high in concentrates (forage:concentrate (F:C) ratio of 19.4:80.6) and the effects of increasing supplementation levels of *C. citratus*, i.e., 0%, 2%, 3% and 4% of the daily DMI, on enteric CH_4_ emissions from beef cattle fed a total mixed ration (TMR) with an F:C ratio of 50.7:49.3. The doses of the supplemented plants were based on the antimethanogenic effects observed in previous experiments. For example, the dose of 365 g DM/d recommended by Hernández-Pineda et al. [18] was used for CB because a significant reduction in daily CH_4_ production at this dose was reported by the authors. The dose of 100 g DM/d utilized by Wanapat et al. [14] was used for CC because at this dose, the authors observed a significant reduction in CH_4_ production, and no information on the in vivo use of MC was found in the literature, so the same dose used for CB was applied based on the similar total tannin (TT) contents of the plants. We hypothesized that these herbs maintain in vivo the antimethanogenic effects observed in in vitro studies.

## 2. Materials and Methods

The study was carried out at the Laboratory for Research on Livestock, Environment and Renewable Energy of the Faculty of Veterinary Medicine and Animal Science (LABRELE) of the Universidad Autónoma del Estado de México in Toluca, Mexico, located at 19°24′15″ N and 99°41′06″ W and 2632 m above sea level.

### 2.1. Experimental Procedures

Two experiments following a 4 × 4 Latin square design were conducted. Experiment 1 took place from 5 January to 29 May 2019. Experiment 2 was carried out from 25 September 2019, to 23 January 2020. All animals received humane care, and the experiments were authorized by the Institutional Subcommittee for the Care and Use of Experimental Animals, protocol DC2018/2-8, of the Universidad Nacional Autónoma de México. Before the start of each experiment, all the animals were dewormed with L-Vermizol^®^ product, and were found to be clinically healthy.

### 2.2. Experiment 1

Eight Charolais × Brown Swiss steers with a 350.4 ± 67 kg live weight (LW) and 18 months of age were distributed in a replicated 4 × 4 Latin square experimental design. The animals were randomly assigned to each of four treatments. The experiment lasted 142 days, and the first 30 days were used for adaptation to a diet high in concentrates. The remaining 112 days were divided into four experimental periods of 28 days each. The DMI and apparent digestibility of DM (DigDM) were measured daily while the animals were in the respiration chambers. A long adaptation period was implemented to prevent the occurrence of ruminal acidosis and any other effects on animal welfare due to the high concentrate level in the diet. The F:C ratio of the CO diet was 19.4:80.6 on a DM basis. In the first two weeks of the adaptation period, the animals were offered a TMR, which contained an F:C ratio of 50:50 (on a DM basis). In week three, the F:C ratio was changed to 25:75, and in the last week, the CO diet was offered. The diet adaptation period was considered adequate, and it was within the 10-to-14 day range suggested by Cochran and Galyean [21] for adaptation to a new diet. During this period, the animals were also adapted to the respiration chambers, they were taken twice per week to the chambers, where they were offered feed and water ad libitum. Each animal stayed inside the chambers for 8 to 10 h/d so that their DMI would not be affected during the assays. In total, each animal visited the chambers for eight days during the adaptation period. For each experimental period of 28 days, the first 21 days were used for adaptation to the experimental diet (treatment), and the remaining seven days were used for sampling (sampling period). Dry matter intake was measured from day 1 (the first day of the sampling period) to day 4. On day 5, the animals were taken in pairs to the respiration chambers to measure CH_4_ emission for 48 h (one animal per chamber), with the experimental periods two days apart.

#### 2.2.1. Treatments

Three herbs (treatments) supplemented at low doses were evaluated in addition to the CO diet. The CO diet (no herbs) was formulated to meet the metabolizable energy and protein requirements of the animals according to the Agricultural and Food Research Council (AFRC) [22]. The CO diet consisted of a TMR offered *ad libitum* and composed (on a DM basis) of 9.7% alfalfa hay, 9.7% oat hay, 5.7% soya bean meal, 68% steam-flaked corn, 4.9% molasses, and 1.9% protected fat (Palmalife^®^, 100% African palm) to prevent hydrolysis in the rumen and potential antimethanogenic effects. Four treatments were evaluated: (1) control diet (CO), (2) CO + 365 g dry matter (DM)/d CB, (3) CO + 365 g DM/d MC, (4) CO + 100 g DM/d CC. Each steer received each treatment once during each of the four periods.

#### 2.2.2. Herb Preparation

CB, an annual Asteraceae herb native to Mexico, was harvested in the Toluca Valley from August to September 2018. CC and MC were purchased from the local market, always from the same supplier. All the herbs were dried for a period of 8 weeks by laying them over metal mesh rack dryers placed 1 m above the floor at an ambient temperature of 22 °C, with a relative humidity of 25%, ample air circulation and no direct sunlight to prevent the denaturation of phenolic and other plant secondary compounds [23]. Once dried, the herbs were ground with a portable hammer mill equipped with 0.58-cm mesh (Bison MMRB-20 model, Aguascalientes, Mexico) and incorporated into the TMR to ensure that the animals would eat the entire daily herb ration provided. This is because the aim of the experiment was to evaluate the effects of the whole plants and not only the effects of their constituent phenolic compounds, essential oils (EOs) and other secondary metabolites. CC was purchased in two different batches, the first batch was purchased in October 2018 for Experiment 1. The second batch was purchased in July 2019 and was used for Experiment 2. Samples of the experimental plants were collected for later chemical analysis in the laboratory.

#### 2.2.3. Measurements and Sampling of Feed and Stool

The DMI, DigDM, and CH_4_ emissions of each animal in the respiration chambers were measured on days one and two. The feed offered on day one was weighed before the start of the assay, and the feeding time was always the same, at 10:00 am. A sample of 5% of the feed offered to each animal was taken daily, and composite samples were formed. Each composite sample of ~1 kg (fresh) was weighed, placed in a paper bag, identified, and dried in a forced-air oven at 60 °C for 72 h to determine its DM content [24]. The next day, before the morning feeding, the chambers were opened, and the ort from each animal was collected directly from the feeder, weighed, and sampled during the two days in the respiration chambers. The DMI/d was calculated as the feed offered minus the ort, both on a DM basis. The composite feed samples were stored in paper bags, identified and reserved for later laboratory analyses. The total production of feces by each animal in 24 h within the chambers was measured daily (two days). Most of the feces (approximately 95%) were collected in a stool container located under the floor of the metabolic cage inside the respiration chamber. This container (1.20 m × 1.05 m) was covered with a wire screen, which separated feces and urine. The feces attached to the walls of the chamber and floor of the cage were removed and collected with a shovel. All feces collected in this way was placed in the bucket and weighed to determine apparent DigDM. A fecal sample of ~10% of the total feces produced by each animal was collected directly from the bucket, weighed, and dried in a forced-air oven at 60 °C for 120 h to determine its DM content [24]. Once dried, a composite sample was formed for each animal, placed in a paper bag, identified and reserved for later laboratory analyses. The animals were weighed weekly. At the same time, on days 1, 8, 15, and 22 of each experimental period, after the animals were fasted for 14–16 h, these four data points plus the starting weight data of the following experimental period were used to calculate the average daily LW gain (ADWG) per animal per experimental period.

#### 2.2.4. Measurement of Methane Emissions

The LABRELE is equipped with two whole-animal open-circuit respiration chambers, as described in Canul-Solis et al. [25]. We used these chambers to measure methane emissions from cattle for 48 h, with one animal per chamber and two animals per run. The respiration chambers were designed following the principle of open-circuit indirect calorimetry [26,27] and were built following the design of Canul-Solis et al. [25]. The chambers were operated and calibrated as described by the previous authors. Briefly, before each assay, two calibrations of the chamber were performed: zero calibration using high-purity nitrogen (N_2_) (Praxair Inc., Toluca, Mexico) and calibration against a reference gas, known as span gas. The N_2_ used for zero calibration was first passed through a drying unit to remove moisture and then through the methane analyzer at a flow rate of 0.3 L/min until a zero reading was obtained. Span calibration was performed using a gas mixture with a known CH_4_ concentration (1000 ppm CH_4_ in high-purity N_2_). The span gas was passed through the methane analyzer (model MA-10, Sable Systems International, Las Vegas, NV, USA) at a rate of 0.3 L/min to obtain a stable reading corresponding to the concentration of CH_4_ in the span gas. The released methane volumes were kept constant by adjusting (10 psi) pressure regulators (Concoa 109–6504) to a controlled flow of 0.2 LPM by means of a flowmeter. The background CH_4_ concentration was verified by injecting ambient air into the analyzer. The air samples were taken with a vacuum pump (PADIIVI.021, APT Instruments, Rochester, NY, USA) at the point of entry of air into the respiration chambers and were found to be negligible. The readings generated by the CH_4_ analyzer were sent to a computer by means of a universal interface (Model UI2, Sable Systems International, Las Vegas, NV, USA), and the data were analyzed with ExpeData software (v.1.9.11. Sable Systems International, Las Vegas, NV, USA). The data from the methane analyzer were recorded and transferred to the computer in real time, and the concentration of CH_4_ in the air coming from the chamber was measured every second. All the data from the 48 h measurement were used to calculate daily CH_4_ emissions. Before the beginning of the experiment, a CH_4_ recovery test was conducted as described by Arceo-Castillo et al. [28] for the types of chambers used in the present experiment, and a 100 ± 2% recovery rate was found.

The *Ym* factor was calculated according to the Tier 2 level method for national inventories calculation of the IPCC [29], this calculation is based on the quotient of the energy lost in the form of methane per animal per day by the total gross energy intake of the same animal per day. The IPCC [2006] assumes that the energy loss as methane equals 55.22 MJ/kg CH_4_ [30].

#### 2.2.5. Chemical Analysis of the Feed and Stool

Before the laboratory analyses, the diet and fecal composite samples were dried in a forced-air oven at 60 °C for 72 h, ground, and passed through a 1-mm sieve. The DM and organic matter (OM) contents were determined according to the procedures of the Official Methods of Analysis [24]. The neutral (NDF) and acid detergent fiber (ADF) contents of the diet and stool samples were determined according to Van Soest et al. [31], heat-stable α-amylase was used for the NDF analyses of the diet and fecal samples. The gross energy (GE) content of the feces and feed was determined with an adiabatic bomb calorimeter (Parr Instrument Company, Moline, IL, USA). The N content in the diet was determined according to the Kjeldahl method [24] and subsequently multiplied by a factor of 6.25 to obtain the protein content. The concentration of total phenols in the experimental herbs was determined with the Folin-Ciocalteu method, the tannin content was evaluated according to the polyvinylpolypyrrolidone method as described in Makkar et al. [23], and the condensed tannin content was assessed with the vanillin method as in Price et al. [32]. The chemical composition of the experimental diets and the polyphenol and tannin contents of the herbs are shown in Table 1.

### 2.3. Experiment 2

A different control (CO) diet was used in this experiment due to animal welfare issues because four of the same animals used in the previous experiment were also used in this experiment and thus were exposed for a long time to the finishing diet. It is well established that beef cattle receiving diets high in concentrates suffer from parakeratosis-rumenitis, a liver abscess complex with a plethora of systemic manifestations [33]. The F:C ratio of this CO diet was 50.7:49.3 on a DM basis, and the diet was composed of 10% alfalfa hay, 20.1% oat hay, 20.6% maize stover, 9.1% soybean, 23.4% ground corn, 3.3% molasses and 13.4% bakery byproducts (cookie waste). The CO diet (no CC) was formulated to meet the metabolizable energy and protein requirements of the animals according to the AFRC [22]. We also used a Latin square experimental design and the same experimental procedures as described in Experiment 1. The four adult F1 Charolais × Brown Swiss steers with a 458 ± 59 kg LW and 26 months of average age were randomly assigned to each of four treatments. The treatments consisted of the (CO) diet with no CC, CO diet + 2% CC (2% CC), CO diet + 3% CC (3% CC) and CO diet + 4% CC (4% CC). The chemical composition of the control diet and the polyphenol and tannin contents of the CC used in this experiment are also shown in Table 1.

This experiment had a duration of 119 days, and the first two weeks were used to allow the animals to adapt to the experimental diet. The animals were already adapted to the chambers. The remaining 105 days were divided into four experimental periods of 26 days each. The first 19 days of each period were used for diet adaptation, days 20 to 26 constituted the sampling period, in which we recorded DMI, CH_4_ emissions, and DigDM. Fecal and diet samples were collected as described before. The weighing and determination of ADWG were carried out as in the previous experiment. The methane production was measured for 48 h on days 24 to 26.

### 2.4. Statistical Model

The experimental data for Experiment 1 were analyzed with analysis of variance on the basis of a replicated Latin square experimental design, as shown in model 1. For Experiment 2, the same model was used but without the effect of the square,

(1) $$Y_{ijkl}\;=\mu \;+\;A_{i}\left(l\right)+\;T_{j}\;+\;P_{k}+S_{l}\;+\;\xi_{ijkl}$$

where *Y_ijkl_* is the response variable of the *i*th animal (*i* = 1, 2, 3, 4) nested in the *l*th square (*l* = 1, 2) that received the *j*th treatment (*j* = 1, 2, 3, 4) during the *k*th period (*k* = 1, 2, 3, 4), *μ* is the overall mean of all observations, *A*_*i*(*l*)_ is the random effect of the experimental animal nested in the *l*th square, *T_j_* is the fixed effect of the treatment, *P_k_* is the fixed effect of the period, *S_l_* is the fixed effect of the square, and *ξ_ijk_* is the random error component. The residuals and random effects were assumed to be independent and normally distributed with a mean of zero and constant variance.

### 2.5. Analysis of the Results

Post hoc pairwise comparisons were conducted using Tukey’s HSD test with the lsmeans function in the lsmeans package of R [34]. In Experiment 2, orthogonal polynomial contrast analyses were used to determine whether the effect of CC on variables that were statistically significant was linear, quadratic or cubic. The analytical procedures for the analysis of variance were performed using the lmer function of the lme4 package [35] in R software [36]. For the polynomial contrasts, we used JMP v11.0.0 statistical software [37].

## 3. Results

Table 2 shows the effects of experimental herb supplementation on DMI, GE intake (GEi), the digestibilities of DM (DigDM), NDF (DigNDF), ADF (DigADF) and GE (DigGE); and the variables related to the enteric CH_4_ emissions in Experiment 1. There were no significant effects (*p* > 0.05) on DMI, DigDM, DigNDF, DigADF, DigGE or average daily CH_4_ production. However, significant differences (*p* < 0.05) in methane yield (g of CH_4_/kg of DMI), ADWG, the methane conversion factor known as the *Ym* factor (energy of CH_4_ as a percentage of GEi), and CH_4_ emission intensity (g of CH_4_/kg of ADWG) were observed. The lowest CH_4_ yields (*p* < 0.05) were observed in the CC and CB treatments, where they were 33% and 28% lower than those in the CO treatment, respectively. The lowest CH_4_ emission intensity (g of CH_4_/kg of ADWG) was observed in the CB treatment, followed by the CC treatment; similarly, the smallest values of the *Ym* factor were observed in the CC and CB treatments, where they differed significantly (*p* < 0.05) from those in the CO and MC treatments. Supplementation with CB resulted in a significantly higher (*p* = 0.03) ADWG than that observed in the other treatments. The third highest LW gain was observed in the CC treatment, but this value was not significantly different (*p* > 0.05) from that obtained under the CO treatment.

Table 3 shows the results for Experiment 2, where increasing supplementation levels of CC were evaluated. No significant differences were observed for DMI, ADWG, CH_4_ yield, *Ym* factor or CH_4_ emission intensity (*p* > 0.05). Significant declines (linear *p* < 0.05; quadratic *p* < 0.05) in total daily CH_4_ production were observed at the 2% and 3% CC supplementation levels, and a numerical reduction was observed at the 4% inclusion level, with 26%, 26.2% and 15% less CH_4_ produced, respectively, than in the CO treatment. Numerical differences were observed for CH_4_ yield at the 2% and 3% supplementation levels, where the yield was 12% and 15% less, respectively, than that in the CO treatment. A significant reduction (linear *p* < 0.05; quadratic *p* = 0.04) in DigDM at the 3% CC inclusion level was observed, but no effects were observed at the other two levels for this variable. However, DigNDF and DigADF declined as the supplementation level of CC increased in comparison with the CO treatment (*p* < 0.05). The declines in DigNDF (linear *p* = 0.02; quadratic *p* = 0.002) and DigADF (linear *p* = 0.02; quadratic *p* = 0.01) at 3% CC were accompanied by a significant reduction (*p* < 0.05) in total daily methane emissions in g/d, suggesting that the CC effect on CH_4_ production was dose-dependent. Therefore, the 2% CC treatment reduced total methane emissions by 26% without affecting DigDM or ADWG. In contrast, the reduction in total daily CH_4_ emission at the 3% CC supplementation level was associated with decreases in the digestibility of DM, NDF and ADF. However, this pattern was not repeated at the 4% inclusion level because supplementation with CC at this level reduced total daily CH_4_ emissions only numerically, with no effect on DigDM (*p* > 0.05) We observed numerical declines (*p* > 0.05) in the *Ym* factor in association with supplementation of CC, particularly at the 2% (*Ym* = 5.9) and 3% (*Ym* = 5.8) inclusion levels, in comparison with the CO treatment (*Ym* = 7.0) (Table 3).

## 4. Discussion

### 4.1. Methane Production

The CH_4_ yield values obtained with the CO diet in Experiment 1 were similar to those reported in the literature, for example, van Lingen et al. [7] developed an intercontinental enteric CH_4_ production database with 1021 individual animal records from beef cattle and calculated important variables such as the average daily emissions (g/d/animal) and CH_4_ yield (g of CH_4_/kg of DMI). The average yield of 15.2 ± 4.2 g of CH_4_/kg of DMI for diets low in forage (<18% forage) reported by these authors is similar to the CH_4_ yields reported in Table 2. Similarly, the CH_4_ yield factor from Experiment 2 (Table 3) is similar to the 20.7 ± 4.7 g of CH_4_/kg of DMI reported by van Lingen et al. [7] for beef cattle diets with more than 25% forage. The lower CH_4_ yield observed in Experiment 1 for the CC and CB treatments in comparison with the CO treatment and the low total daily emissions observed in Experiment 2 suggest that these herbs reduced enteric CH_4_ emissions in terms of CH_4_ yield and daily emission in g/d, respectively. The total daily emission under CC supplementation was also numerically smaller (<16%) than that under the CO diet in Experiment 1, and 26% lower than that under the CO diet in Experiment 2 (*p* < 0.05), where a quadratic response (*p* < 0.05) to CC supplementation was also observed. The decreased CH_4_ yield (*p* < 0.05) in Experiment 1 is partially the result of a numerical increase in DMI in the CB and CC treatments. However, this reduction in CH_4_ yield cannot be solely explained by the increased DMI because there is sufficient evidence that CH_4_ yield is not correlated or is poorly correlated with DMI [38,39]. Thus, lower methane yields not necessary is the result of higher DMI as other mechanisms are involved. According to Herd et al. [38], CH_4_ yield is not correlated (r ± SE) with DMI (−0.02 ± 0.04). Similarly, Benaouda et al. [39] reported a correlation coefficient for DMI and CH_4_ yield of only −0.133. Herd et al. [38] conducted a study to evaluate a number of methane measures that target CH_4_ production independent of feed intake and to examine their phenotypic relationships with growth and body composition. The authors collected data from 777 young Angus bulls and heifers that were fed a roughage diet and measured for CH_4_ production in open-circuit respiration chambers for 48 h. They concluded that reducing CH_4_ production per se can have a negative impact on the growth and body composition of cattle due to decreased DMI. Reducing CH_4_ yield, however, will likely reduce CH_4_ production without impacting productivity. Further, they reported that CH_4_ yield was not correlated with DMI but was positively and strongly correlated with CH_4_ production. This implies that reducing CH_4_ yield as a GHG mitigation strategy will have no impact on DMI and hence maintain animal productivity but have the correlated effect of reducing CH_4_ production, like in our Experiment 1.

The lack of a significant difference in total daily methane emission between the CC and CO treatments in Experiment 1 could also be explained by the high concentrate content of the basal diet. According to Van Kessel and Russell [40], diets high in concentrates reduce ruminal pH, and as methanogens are acutely pH-sensitive, it has been suggested that diets that are capable of reducing ruminal pH could serve as a practical means to achieve reductions in enteric CH_4_ production. Thus, it is believed that at the low F:C ratio used in Experiment 1, methanogenesis was significantly reduced, with less potential for further reductions due to CC supplementation, a similar response was reported by Lovett et al. [41] with the use of coconut oil. This pattern was not observed in Experiment 2 because the potential for methane reduction was larger due to the higher forage content of the basal diet used in this experiment. Results for the *Ym* factor in Experiment 1 seem to support this hypothesis because the observed *Ym* values are similar to those reported for diets with a high inclusion of concentrates. For example, van Lingen et al. [7], reported an average *Ym* value of 4.5 ± 1.2 (n = 139) for diets low in forage (≤18% forage). Our *Ym* value for the CO treatment in Experiment 1 (*Ym* = 5.0) is within the range reported by these authors. However, the highly significant lower *Ym* values observed for the CC and CB treatments in comparison with the CO treatment could be explained by the antimethanogenic effect of the condensed tannin in the experimental herbs despite numerically higher DMI and GEi observed in these treatments. On the contrary, van Lingen et al. [7] reported an average *Ym* value of 6.3 ± 1.4 (n = 882) for diets higher in forage (≥25% forage), which is similar to the *Ym* = 7.0 obtained in the present work. Thus, increasing levels of CC supplementation in Experiment 2 seem to explain the decrease in the *Ym* factor because the *Ym* factor passed from 7.0 in the CO treatment to 5.8 in 3% CC treatment; meaning that less energy from the feed was lost as CH_4_. However, this difference was only numerical, so more research is necessary to clarify the role of CC on energy partitioning in the animal. Our results are similar to those reported by Lovett et al. [41], in a study conducted with 36 finishing heifers to investigate the effect of coconut oil on methane production. They evaluated six experimental diets, with different F:C ratios, 65:35, 40:60, and 10:90, supplemented with two levels of coconut oil (0 or 350 g/d). They found that the *Ym* factor decreased from 6.06 to 4.44 as the inclusion level of forage in the diet decreased from 65% to 10%, with a quadratic effect, as in the present work. These authors also observed that coconut oil levels of 0 g/d and 350 g/d resulted in *Ym* factors of 6.60 and 4.83, respectively, which represent a decrease of 26% in the *Ym* factor. In our Experiment 1, we found that *Ym* factor decreased by 28 and 32% due to the supplementation of CB and CC, respectively, compared to the CO diet.

The reduction in CH_4_ production could also be attributed to the high condensed tannin content, particularly in the CC diet (Table 1). Several studies have demonstrated that the antimethanogenic activity of phenolic compounds can be attributed to condensed tannins in plants [42,43]. According to Bhatta et al. [44], tannins suppress methanogenesis directly by reducing the methanogenic population in the rumen or indirectly by reducing protozoa. The symbiosis between methanogenic archaea and protozoa in the rumen is well established [45]. According to Kazunari [46], ciliated protozoa are the principal component of the rumen microbiota because they contribute up to 50% of the biomass in the rumen [47] and significantly contribute to the digestion of ruminants [46]. As anaerobic fermentative microorganisms, rumen ciliated protozoa produce a significant amount of hydrogen (interspecies hydrogen transfer between protozoa and archaea by which both can grow faster) and formate. Methanogenic archaea are closely associated with ciliated rumen protozoa [48] and therefore with CH_4_ emission. Protozoa produce butyrate and acetate, two VFAs whose production releases 2 and 4 moles of H_2_, respectively, per mole of fermented glucose, half of this H_2_ is used by methanogenic archaea [49]. This group of bacteria is usually found on the surface of (ectosymbiosis) or inside (endosymbiosis) protozoa, benefiting from the H_2_ released by protozoa and producing CH_4_, the main source of energy necessary for the growth of methanogenic archaea [50]. Condensed tannins can also reduce methane emissions by indirectly decreasing H_2_ production as a result of decreased fiber digestion by protozoa [51]. This could be observed in Experiment 2, where DigDM, DigNDF, and DigADF were reduced by including CC in the basal diet. Moreover, this effect is in line with that reported by Newbold et al. [47], who mentioned that eliminating protozoa from the rumen significantly decreased rumen OM digestibility (−7%), particularly NDF (−20%) and ADF (−16%) digestibility, probably because of the loss of protozoal fibrolytic activity. Unfortunately, in the present work, we did not measure the effect of CC on the protozoa in experimental animals. However, our results suggest that the condensed tannins in CC probably affected the population of ruminal protozoa, and thus fiber degradation and, as a consequence, CH_4_ production were also reduced. Previous studies conducted by our group indicated that the tannins in *C. bipinnatus* can reduce enteric CH_4_ yield in dairy cattle fed a diet with an F:C ratio of 62:38 by 16% in comparison with that in a control diet [18]. Similarly, Gomaa et al. [17] demonstrated that the inclusion of CC at a 10% level reduced in vitro CH_4_ production by 14.5%.

There have been few studies on the effects of CC on enteric CH_4_ production. Wanapat et al. [15] conducted an in vitro study to evaluate the effects of supplementing the diet with different levels of powdered CC (0, 100, 200 and 300 g of dry CC/d) on rumen ecology, rumen microorganisms and the digestibility of nutrients in beef cattle and found that the protozoal population significantly declined from 6.3 × 10^6^ to 4.6 × 10^6^ regardless of the CC dose. Wanapat et al. [15] also reported that the supplementation of CC powder at 200 and 300 g/d decreased bacterial populations relative to those under 100 g/d of CC powder, possibly due to decreases in gram-positive bacteria, because gram-positive bacteria appeared to be more susceptible than gram-negative bacteria to inhibition by plant EOs compounds. In a similar study, Wanapat et al. [14] showed that CC alone (100 g DM/d) or in association with peppermint (10 g DM/d) and garlic (40 g DM/d) reduced the communities of protozoa and bacteria; CC also reduced DM digestibility and CH_4_ emissions in beef cattle. However, it is important to stress that Wanapat et al. [14] estimated CH_4_ production and did not measure it. Some in vitro studies have also shown that *Cymbopogon* species can affect protozoan populations and CH_4_ production. For example, Bhatta et al. [9] conducted a study to evaluate the potential of secondary plant metabolites from 38 sources to serve as antimethanogenic additives in ruminant diets. They found that *Cymbopogon martinii* reduced the protozoan population by up to 19% and CH_4_ production by 4.5%, even when the condensed tannin content of the plant used was negligible. Therefore, this reduction may be attributed to other secondary metabolites present in CC, such as EOs or hydrolysable tannins (HTs = 1.58 g/kg DM). The reduction in fiber digestion was more clearly observed in Experiment 2 at all supplementation levels.

Total daily CH_4_ production and CH_4_ yield were higher in Experiment 2, this was expected because the F:C ratio of the basal diet in the former was 50.7:49.3. It is well established that both daily CH_4_ emission and CH_4_ emission per unit of DMI increase with forage content in the diet as a result of increased NDF intake [52], provided that the forage is sufficiently digestible [53]. Thus, the reductions in CH_4_ production associated with the 2% and 3% CC supplementation levels in this experiment could be consequences of a reduction in the digestibility of fiber in the diet. This reduction in CH_4_ production could (partially) be attributed to the high polyphenol and tannin contents in the CC used, which may have affected fiber-degrading bacteria and protozoa. For example, the polyphenol content of CC (7 g/kg DM) used in Experiment 2 (Table 1) was almost twice that (2.6 to 3.8 g/kg DM) reported by Thorat et al. [54]. Similarly, the TT content (9.9 g/kg DM) of our CC was higher than that (6 g/kg DM) reported by Avoseh et al. [55]. Moreover, the TT content of the CC used in Experiment 2 was three times higher than that used in Experiment 1 (Table 1). In contrast, the condensed tannin (CT) content in the CC used in Experiment 2 was lower than that in Experiment 1. However, the total daily intake of CT by animals in Experiment 2 was more than two times higher than that in Experiment 1 because of the higher CC intake, e.g., 195 g DM/d for the 2% CC treatment.

The antimethanogenic activity of CC could also be explained by EOs contained in this plant, particularly citral (3,7-dimethyl-2,6-octadien-1-al) [56]. According to Patra et al. [51], methanogens may be directly or indirectly inhibited by EOs via the inhibition of protozoa and H_2_-producing bacteria in the rumen. CC EOs have been found to contain up to 75–85% citral [57]. Many EOs have dose-dependent effects on bacteria, protozoa, and fungi [58]. For example, Joch et al. [59] investigated the effects of 11 active compounds of EOs (1000 μL/L of diluted rumen fluid), such as eugenol, carvacrol, citral, limonene, 1,4-cineole, p-cymene, linalool, bornyl acetate, α-pinene, and β-pinene, on rumen fermentation and CH_4_ production and found that citral reduced CH_4_ production by 44% and limonene by 23%; both compounds are present in CC EOs. Similarly, Ram Kumar et al. [60] conducted a study to investigate the in vitro rumen fermentation profiles of two different diets, namely, oat hay only or a mixture of oat hay and concentrate in a 60:40 ratio supplemented with graded levels (0.0, 10, 20, 40 and 80 µL/40 mL of buffered rumen liquor) of CC EOs, in buffered buffalo rumen inoculum. They found that the CH_4_ concentration in the headspace gas decreased linearly with an increasing concentration of CC EOs irrespective of diet and that the reduction ranged from 31% to 100% at the lowest and highest doses of CC EOs, respectively.

However, more research on the use of CC to reduce enteric methane emissions is necessary because the concentrations of EOs and polyphenols in CC can change with various factors, such as geographic location [61], cultivation method, harvesting time, controlled oxidation, and withering conditions [16]. This may explain why some authors have not found any effects of CC in reducing enteric methane emissions, for example, Nanon et al. [62] reported no effect of CC oil supplementation (200 mg/kg diet) to a diet consisting of forage and concentrate on DM degradability or CH_4_ production when using a rumen simulation technique. Therefore, it is suggested that those who wish to replicate the present study select an appropriate *Cymbopogon* species and harvest it when the concentrations of polyphenols and EOs are the highest. Chemical analyses are necessary to confirm the appropriate concentration of secondary metabolites before the beginning of the experiment. This requisite is relevant because it is well established that the concentrations of EOs and tannins in CC vary throughout the year [63]. Otherwise, it may be possible to obtain a result different from that described in our work. Finally, careless handling of CC may lead to the loss of its critical antimethanogenic components, which emphasizes the importance of adequately processing and preserving CC before using it in similar research.

Other compounds present in CC, such as flavonoids, can affect ruminal CH_4_ production. Quercetin and kaempferol are two flavonoids found in CC in significant quantities [55], and according to Oskoueian et al. [20], flavonoids can modulate rumen fermentation by selectively reducing VFAs production, DigDM and CH_4_ production. Thus, the addition of kaempferol (in its pure form) significantly reduced the populations of almost all of the rumen microorganisms in in vitro assays [20]. Thus, a reduction in CH_4_-producing microorganisms is reflective of a decrease in CH_4_ production. In contrast, quercetin and naringin at a concentration of 4.5% (*w*/*w*) of the substrate suppressed in vitro CH_4_ production and decreased rumen protozoa and methanogen populations without affecting DigDM and other fermentation parameters. Apparently, flavonoids such as kaempferol affect the activity of microbial enzymes such as xylanase and carboxymethylcellulase, which are involved in the degradation of hemicellulose (DigNDF) and cellulose (DigADF), respectively. However, more research is necessary to evaluate the effects of CC flavonoids on in vivo ruminal CH_4_ production. Thus, the effect of CC on reducing rumen CH_4_ production may also be the result of the joint action of CTs, flavonoids, and EOs on rumen microbes [20].

### 4.2. Digestibility, Dry Matter Intake, and Live Weight Changes

The effects of herbs on ADWG need to be evaluated in a further study. The short experimental periods in the current study were insufficient to obtain reliable ADWG results; in this regard, the present ADWG results should be considered preliminary and interpreted with caution. We are also cautious with regard to the DMI results, as large numerical differences could not be identified as statistically significant. Significant reductions in DigDM, DigNDF, and DigADF were observed in Experiment 2 at the 3% CC inclusion level, which were associated with a reduction in LW gain. A similar effect on digestibility was reported by Ram Kumar et al. [60], where truly degradable DM was reduced in all the treatments except that with a low level of CC oil in both dietary substrate groups. The reductions in DigDM, DigNDF, and DigADF at relatively high doses of CC could be due to the inhibition of fiber-degrading bacteria. This can explain the low DigNDF and DigADF in diets with different levels of CC in Experiment 2, which directly affected the digestibility of energy, and therefore the numerically lower DMI when CC was supplemented. Although we did not measure the effect of CC on rumen bacteria, evidence in the literature suggests that gram-positive bacteria are more susceptible to inhibition by plant EOs compounds than are gram-negative bacteria [64]. However, other authors have stated that the major bioactive compounds identified in CC EOs, namely, α-citral (geranial) and β-citral (neral), exhibit antibacterial activity by inhibiting the growth of both gram-positive and gram-negative bacteria [16]. This idea is in line with the finding of Wanapat et al. [14], who reported a reduction in bacterial count upon supplementation of the diet of cattle with CC powder at a dose of 200 g/d but not 100 g/d.

Finally, the results suggest that supplementation with CB reduced the enteric CH_4_ yield and the intensity of CH_4_ emission without affecting the digestibility of the basal diet or the fiber fraction. Furthermore, CB did not affect ADWG because the largest weight gain was observed in this treatment, which was associated with low CH_4_ emission. This result is in line with previous findings reported by Hernández-Pineda et al. [18] and Min et al. [65]. Min et al. conducted a study to determine the effects of quebracho CT supplementation on the in vitro ruminal fluid gas production, in vivo ruminal fluid protein fractions, bloat dynamics, and ADWG of steers grazing on winter wheat. The authors reported an ADWG of 2.09 kg/day for steers supplemented with 1% CT/kg of DMI and grazing on winter wheat. According to Min et al. [65], the combination of increasing bypass protein flow to the small intestine and decreasing frothy bloat and CH_4_ production due to CT likely led to the 15% increase in ADWG observed with CT supplementation of steers grazing on wheat forage. The effect of CB on ADWG needs further evaluation because it may be necessary to use more extended experimental periods than those used in the present work. For example, a completely randomized design with more animals and experimental periods of up to 40 days could be used.

## 5. Conclusions

It was concluded that *Cosmos bipinnatus* and *Cymbopogon citratus* decreased in vivo methane production by beef cattle; the effects on CH_4_ production were dependent on diet and the dose and tannin content of these herbs. On the contrary, no antimethanogenic effect was observed by chamomile. On the other hand, these herb effects on animal performance in both experiments and on digestibility in Experiment 1 should be considered preliminary as more research is necessary. It is also concluded that supplementation of CC at levels above 190 g DM/d can reduce daily CH_4_ production but at the expense of reducing the digestibility of DM, and fiber fractions of diets. To the best of our knowledge, this was the first study in which the response of CH_4_ production to CC supplementation was measured in vivo.

## Figures and Tables

**Table 1:** Chemical composition of the control diets used in Experiments 1 and 2 and polyphenol and tannin contents of the herbs used for Experiments 1 and 2 to reduce enteric methane emissions in beef cattle.

	Control Diet		Control Diet	
	Experiment 1		Experiment 2	
DM (g/kg)	926.5 ± 7.8		963.4 ± 3.5	
OM (g kg/DM)	958.8 ± 7.6		938.4 ± 6.1	
CP (g kg/DM)	149.4 ± 4.9		117.2 ± 7.2	
EE (g kg/DM)	26.5 ± 2.0		15.7 ± 2.3	
CF (g kg/DM)	55.4 ± 5.3		149.0 ± 17.2	
NFC (g kg/DM)	731.9 ± 17.2		656.7 ± 17.6	
GE (MJ kg/DM)	18 ± 0.3		15.6 ± 0.4	
NDF (g kg/DM)	294.2 ± 128.5		426.9 ± 29.2	
ADF (g kg/DM)	76.2 ± 12.7		223.1 ± 24.7	
**Herbs**	***Matricaria chamomilla***	***Cosmos bipinnatus***	***Cymbopogon citratus***	
			**Experiment 1**	**Experiment 2**
TP (g kg/DM)	5.9	9.6	9.5	7.0
TT (g kg/DM)	3.9	3.6	3.3	9.9
CT (g kg/DM)	0.2	2.3	60.7	44.0
EE (g kg/DM)	-	-	1.9	1.9

DM = dry matter, OM = organic matter, CP = crude protein, EE = ether extract, CF = crude fiber, NFC = nonfiber carbohydrates, GE = gross energy, NDF = neutral detergent fiber, ADF = acid detergent fiber, TP = total phenols, TT = total tannins, CT = condensed tannins.

**Table 2:** Effects of *Matricaria chamomilla*, *Cosmos bipinnatus* and *Cymbopogon citratus* supplementation on dry matter intake, digestibility, live weight gain and methane production in F1 beef steers on a finishing diet in Experiment 1.

Treatments					SEM	*p*-Value
	CO	MC	CB	CC		
DMI (kg/d)	7.92	8.66	10.3	9.63	0.93	0.109
LW (kg)	390	373	395	392	21.8	0.196
DMI (%LW)	2.10	2.31	2.60	2.41	0.15	0.159
DigDM (%)	78.5	82.5	82.8	77.9	2.61	0.471
DigNDF (%)	80.6	83.0	79.7	83.2	2.09	0.455
DigADF (%)	64.0	64.2	56.9	59.7	3.56	0.484
DigGE (%)	78.6	84.1	84.4	80.2	2.37	0.210
ADWG (kg/d)	1.43 ^ab^	0.88 ^b^	1.81 ^a^	1.29 ^ab^	0.21	0.029
CH_4_ (g/d)	128	124	118	107	13.30	0.700
CH_4_ (g/kg of DMI)	16.3 ^a^	14.3 ^ab^	11.8 ^b^	11.0 ^b^	1.08	0.009
CH_4_ (g/kg ADWG)	132 ^a^	149 ^a^	67.5 ^b^	103 ^ab^	20.5	0.028
*Ym* (%)	5.02 ^a^	4.41 ^ab^	3.62 ^b^	3.38 ^b^	0.33	0.009
GEi (MJ/d)	142	155	184	173	16.7	0.112
DEi (MJ/d)	113	131	157	143	16.7	0.136

CO = control diet; MC = *Matricaria chamomilla* (365 g DM/d); CB = *Cosmos bipinnatus* (365 g DM/d); CC = *Cymbopogon citratus* (100 g DM/d); DMI = dry matter intake; LW = live weight; DMI (%LW) = dry matter intake as a % of live weight; DigDM = digestibility of the dry matter; ADWG = average daily live weight gain; CH_4_ = methane; CH_4_ (g/kg of DMI) = methane yield; DigGE = digestibility of the gross energy intake; DigNDF = digestibility of the neutral detergent fiber; DigADF = digestibility of the acid detergent fiber; CH_4_ (g/kg ADWG) = intensity of methane emission; *Ym* = methane conversion factor, energy of CH_4_ as a percentage of GEi; GEi = gross energy intake; DEi = digestible energy intake; SEM = standard error of the mean. Values in the same row with different superscripts letters ^a,b^ are significantly different (*p* < 0.05).

**Table 3:** Effects of increasing levels of *Cymbopogon citratus* supplementation on dry matter intake, digestibility, live weight gain and methane production in F1 beef steers fed a total mixed ration in Experiment 2.

Experimental Diets					SEM	*p*-Value	Statistical Significance		
	CO	2% CC	3% CC	4% CC			L	Q	C
DMI (kg/d)	16.0	14.0	13.7	14.3	1.41	0.666	NS	NS	NS
LW (kg)	508	507	513	511	3.41	0.647	NS	NS	NS
DMI (%LW)	3.16	2.81	2.69	2.79	0.24	0.602	NS	NS	NS
DigDM (%)	76.2	69.9	65.5	72.2	2.46	0.050	0.05	0.04	NS
DigNDF (%)	71.8	65.2	54.9	66.6	1.73	0.003	0.02	0.002	0.02
DigADF (%)	71.1	63.8	56.0	64.7	1.86	0.007	0.02	0.01	NS
DigGE (%)	77.7	73.1	66.9	76.6	1.25	0.003	NS	0.001	0.02
ADWG (kg/d)	1.16	1.01	1.10	1.20	0.92	0.561	NS	NS	NS
CH_4_ (g/d)	308	228	227	261	22.9	0.050	0.05	0.05	NS
CH_4_ (g/kg of DMI)	20.0	17.6	16.9	19.9	1.21	0.262	NS	NS	NS
CH_4_ (g/kg ADWG)	291	230	236	252	29.5	0.511	NS	NS	NS
*Ym* (%)	7.03	5.94	5.83	6.60	0.38	0.197	NS	NS	NS
GEi (MJ/d)	252	219	215	224	22.1	0.656	NS	NS	NS
DEi (MJ/d)	197	161	144	172	17.5	0.285	NS	NS	NS

CO = control diet; CC = *Cymbopogon citratus* inclusion level expressed on a DM basis; DMI = dry matter intake; LW = live weight; DMI (%LW) = dry matter intake as a % of live weight; DigDM = digestibility of the dry matter; ADWG = average daily live weight gain; CH_4_ = methane; CH_4_ (g/kg of DMI) = methane yield; DigGE = digestibility of the gross energy intake; DigNDF = digestibility of the neutral detergent fiber; DigADF = digestibility of the acid detergent fiber; CH_4_ (g/kg ADWG) = intensity of methane emission; *Ym* = methane conversion factor, energy of CH_4_ as a percentage of GEi; GEi = gross energy intake; DEi = digestible energy intake; SEM = standard error of the mean. L, Q, and C: linear, quadratic and cubic effects, respectively.
